# Species richness and biogeographical affinities of the marine molluscs from Bahía de Chamela, Mexico

**DOI:** 10.3897/BDJ.8.e59191

**Published:** 2020-12-11

**Authors:** Eduardo Ríos-Jara, Cristian Moisés Galván-Villa, María del Carmen Esqueda-González, Manuel Ayón-Parente, Fabián Alejandro Rodríguez Zaragoza, Dafne Bastida-Izaguirre, Adriana Reyes-Gómez

**Affiliations:** 1 Departamento de Ecología, Universidad de Guadalajara, Zapopan, Mexico Departamento de Ecología, Universidad de Guadalajara Zapopan Mexico; 2 Universidad Pedagógica Nacional, Guadalajara, Mexico Universidad Pedagógica Nacional Guadalajara Mexico

**Keywords:** Mollusca, Bivalvia, Gastropoda, Polyplacophora, Scaphopoda, Cephalopoda, richness, range extension, new records, checklist, biogeography, Tropical Eastern Pacific, Mexican Pacific

## Abstract

For more than 10 years (2007-2018), the benthic macroinvertebrates of Bahía de Chamela (Mexican Pacific) were sampled at 31 sites (0-25 m depth). A total of 308 species of the five main classes of benthic molluscs were obtained (106 bivalves, 185 gastropods, 13 polyplacophorans, two scaphopods and two cephalopods). This is a significant increase in the number of species (246 new records) compared to the 62 species previously recorded more than 10 years ago. The distribution in the 31 localities of the bay is given for the first time for most of the species, together with information on its ecological rarity (incidence in the samples). Two families of bivalves (Veneridae and Mytilidae) and three families of gastropods (Calyptraeidae, Muricidae and Collumbellidae) comprised ~ 30% of all species. Ecological rarity was evident with 45 families (45.0%) with only one species and 178 species (57.8%) collected in one site and 67 (21.8%) in two sites. The molluscs of Bahía de Chamela represent 12.2% of all species recorded in the Mexican Pacific. Their biogeographic affinities are mostly related to the Tropical Eastern Pacific (TEP) including the oceanic islands and a few are restricted to the Tropical Mexican Pacific (TMP). Some have broader distributions to adjacent northern and southern temperate regions of the American Pacific, one to the western Atlantic, two pantropical (PAN) and two cosmopolitans (COS). The range distribution of each species was reviewed and updated, thus finding that seven species have extended their ranges of geographic distribution.

## Introduction

Although molluscs are one of the best-known groups of marine invertebrates in the Mexican Pacific (e.g. Pérez-Peña and Ríos-Jara 1998, Esqueda-González et al. 2000, Esqueda-González et al. 2014, Ríos-Jara et al. 2003, Ríos-Jara et al. 2008, Ríos-Jara et al. 2009, Hermosillo et al. 2006, Bertsch 2008, Bastida-Zavala et al. 2013, Flores-Rodríguez et al. 2014, Hendrickx et al. 2007, Hendrickx et al. 2019, Corgos et al. 2014, Barrientos-Lujan et al. 2017), there are particular areas where studies on molluscs are scarce, amongst which is the Bahía de Chamela. The bay has a very particular location between two oceanic systems, the Gulf of Tehuantepec and the Gulf of California (Kesler 2006) and has a major interest because it was the first marine sanctuary of Mexico, officially declared as a protected area in 2002 and incorporated into the National System of Protected Natural Areas, because of its high biodiversity and heterogeneity of habitats (e.g. islands, islets, an estuary with mangrove swamps, rocky and coral reefs) which are representative of the TMP (LGEEPA 1988). However, when it was established as a protected area, there were only preliminary inventories of the marine fauna; the list of mollusc species was quite scarce with only a few nominal species to recognize the real taxonomic diversity (CONANP 2008).

During recent years, inventories performed in Bahía de Chamela have improved our knowledge of major marine taxonomic groups, including the resident seabirds of the islands and islets (Hernández-Vázquez et al. 2010), the fish assemblages (Galván-Villa 2015, Galván-Villa et al. 2016) and the parasites of several fish species (León et al. 1997, Mendoza and Pérez 1998, Pérez-Ponce de León et al. 1999). Amongst the invertebrates, most of this work has focused on echinoderms (Nuño-Hermosillo et al. 2006, Ríos-Jara et al. 2013) and crustaceans (Bastida-Izaguirre et al. 2013, Ayón-Parente et al. 2014, Ayón-Parente et al. 2016). There are only two previous studies on the marine molluscs performed almost 20 years apart from each other reporting the most conspicuous species with a total of 49 gastropods, 10 bivalves and three chitons (Román-Contreras et al. 1991, López-Uriarte et al. 2009).

Bahía de Chamela is included in the Marine Priority Region 26, which was established as a region of main concern by the National Commission for the Knowledge and Use of the Biodiversity of Mexico (CONABIO); it is also part of the National System of Marine Protected Areas. The area of the protected polygon of the bay covers 1981 ha and includes a series of islands and islets, with the most representative marine environments of the TMP (CONANP 2008). In Mexico, as in other countries, one of the main priorities in protected areas is to guarantee a sustainable management and the conservation of biodiversity. Comprehensive knowledge of marine biodiversity is necessary as part of the baseline information required to propose strategies for the conservation and adequate management of natural resources of the bay, due to the imminent plans to develop touristic resorts that will potentially affect the biodiversity (Miranda et al. 2011). Here, we provide an inventory of benthic molluscs from the bay with information on the local distribution and habitat, ecological rarity, biogeographical affinities and those species with distribution range extensions.

## Material and methods

### Study Site

**Figure 1.**
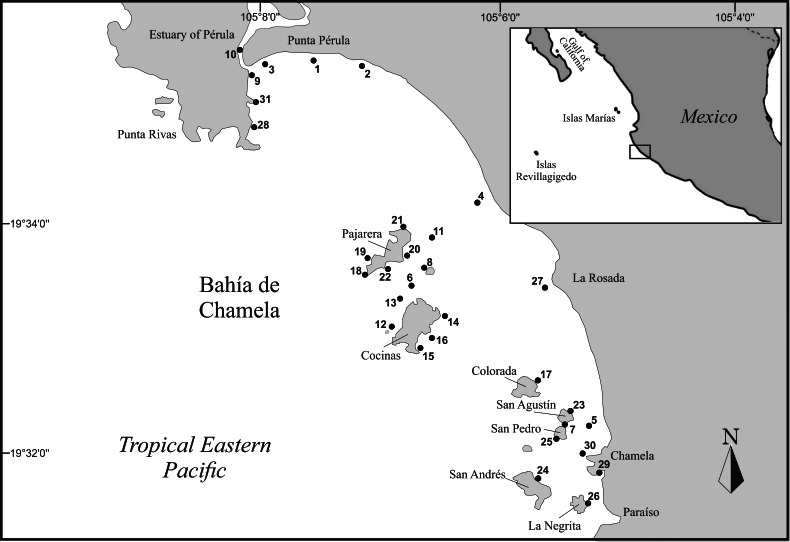
Study area and sampling sites in Bahía de Chamela, Mexico. Name and detailed information on the location, geographic position and depth of each site are reported in Table 1.

Bahía de Chamela is located in the central part of the Mexican Tropical Pacific between Punta Rivas (19°34'36''N, 105°08'33''W) and Punta Chamela (19°30'52''N, 105°04'56''W) (Fig. 1). The bay has a northwest-southeast orientation with a coastline length of approximately 12.3 km. There is a mixed coastal morphology, consisting of small coves and capes, short cliffs interspersed with rocky beaches and long sandy shores. Two main islands and numerous islets are distributed along the bay in a north-south direction mostly parallel to the coastline. The two main islands (Pajarera and Cocinas) are located towards the middle portion of the bay. Pajarera has a longitudinal axis in the northeast-southwest direction, with a maximum height of 60 metres above sea level and a rough and mostly rocky coastline; Cocinas is the larger island, with smoother coastal morphology (maximum height 34 metres above sea level) and circular configuration with small coves and sandy beaches towards the southeast side. There are at least ten small islands and islets in the bay; the most important are San Agustin, San Pedro, San Andrés, Colorada, El Novillo, Los Negritos and Los Anegados.

### Data collection

Collections were made from 31 sites in the bay between 2007 and 2018. Most localities were visited at different times during the warm-rainy and warm-dry seasons of the year for better coverage of the seasonality of the area. The intertidal and adjacent shallow subtidal (0.5–25 m depth) were considered in each site. However, since several small islands and inlets do not have a well-defined intertidal zone, in these sites, sampling was performed only in the subtidal. Sampling was performed through direct search both in the intertidal and in the shallow subtidal during snorkelling and SCUBA diving. In the intertidal zone, molluscs were recorded through a direct search on and beneath the rocks mainly during the low tides. Those found in sandy beaches were obtained by digging and then sieving the sand through meshes of 10 mm. In the shallow subtidal, the molluscs were observed and identified in situ during snorkelling and SCUBA diving. Additionally, a naturalist´s trawling dredge (mesh size = 2.5 cm, cod-end mesh size = 1.3 cm) (English et al. 1997) was used to collect benthic molluscs from soft sandy substrates of several sites (Table 1). To complete the inventory, some rocks and pieces of dead coral were collected from the intertidal and subtidal environments for a better examination in the laboratory in search of the semi-infaunal, endoliths infaunal and epifaunal forms of molluscs. Endolithic specimens (i.e. those growing within rocks or other hard substrates) were obtained by breaking rocks, shells and coral fragments. Epifaunal specimens (i.e. species attached to a hard substrate) were obtained by scraping the surface of rocks. Semi-infaunal specimens (i.e. partially buried in the sediment, but protruding above it) and infaunal specimens (< 0.5 cm) (i.e. those living buried in the soft substrate) were obtained by screening the sediment (Levinton 2001). Photographic records of some live heterobranchs (e.g. sea slugs, sea hares) complement the collection. Only the conspicuous and larger species (> 0.5 cm) collected alive were considered for the inventory (no micro-molluscs).

Taxonomic identification was performed using specialized literature: Keen (1971), Hermosillo et al. (2006), Coan and Valentich-Scott 2012, Kaas and Van Belle (1985), Kaas and Van Belle (1985), Kaas and Van Belle 1987a, Kaas and Van Belle 1987b), Reyes-Gómez 2016, Emerson 1962, Steiner and Kabat (2004). When necessary, additional bibliographic references for particular genera and species were also used. The classification and nomenclature of major clades of gastropods are according to Bouchet et al. (2017); taxonomy was finally reviewed and updated according to Skoglund (1991a), Skoglund (1991b), Skoglund (2001), Skoglund (2002) and the World Register of Species WoRMS on the internet (http://www.marinespecies.org). A reference collection was set up with all the curatorial information in the Marine Biological Collections Area of the Laboratory of Molecular Ecology, Microbiology and Taxonomy at the Department of Ecology (LEMITAX), University of Guadalajara, Mexico. Voucher specimens were also deposited in this laboratory.

### Data analysis

The sampling effort and the completeness of the species inventory were evaluated with sample-based rarefactions, using the number of species per sampling site (Magurran 1988). The expected species richness was estimated with Chao 2 Jackknife 1 and Jackknife 2 with 10,000 randomizations without replacement in EstimateS 9.1 (Colwell 2006). The ecological rarity refers to how frequently the species are recorded in the samples and greatly influences any estimate of the number of species in a given region. Therefore, the unique species are those recorded on a single occasion (sample) and the duplicate species on two occasions (samples) during the entire study (Kunin and Gaston 1993). It then indicates the preference of the species for a certain habitat when there are many other habitats available in the bay. To evaluate this ecological rarity, the accumulation curves of unique and duplicate species were obtained.

The geographic distributions of each species were first reviewed in the specialized literature (e.g. Keen 1971, Hendrickx and Brusca 2005, Hermosillo et al. 2006, Coan and Valentich-Scott 2012, Kaas and Van Belle 1985, Kaas and Van Belle 1985, Kaas and Van Belle 1987a, Kaas and Van Belle 1987b, Reyes-Gómez 2016, Steiner and Kabat 2004) and they were also supplemented or updated using additional bibliographic references for particular genera and species. Biogeographic affinities were then assigned and evaluated, based on the biogeographic regions proposed by Spalding et al. (2007). Biogeographic affinities were established by recognizing eight major realms and their respective biogeographic provinces where the species have been recorded. In the case of the Province TEP, greater detail was considered because it includes most of the species and is where the Bahía de Chamela is located. Then four main ecoregions of this Province are specified: Tropical Mexican Pacific (TMP), Ecoregion Revillagigedo Islands, Mexico (IREV), Ecoregion Clipperton Atoll (ICLI), Ecoregion Coco Island, Costa Rica (ICOC), in addition to Tres Marias Islands, Mexico (ILTM). Finally, PAN and COS species are also noted.

## Results

A total of 308 species of five classes of molluscs were found in Bahía de Chamela (Suppl. material 1). The number of species and percentages of genera, families and orders is presented in Table 2.

**Figure 2.**
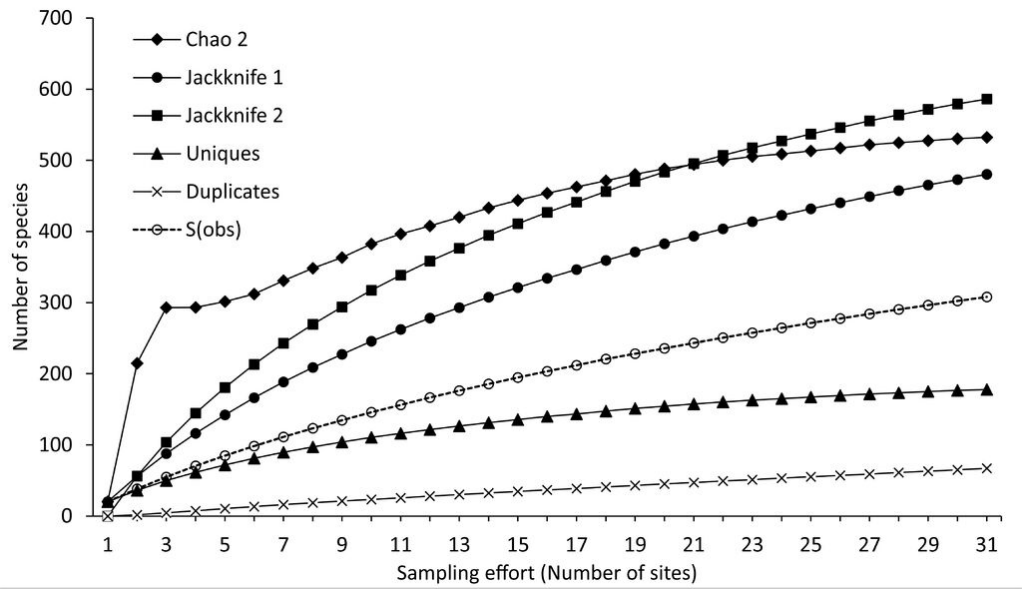
Observed (S) and expected mollusc species based on sample rarefaction curves, with non-parametric indices, unique and duplicate species from sites in Bahía de Chamela, Mexico.

As a result, the species accumulation curves, obtained from sample-based rarefactions, showed a tendency to the asymptote, indicating an acceptable representation of the species richness of Bahía de Chamela, according to the sampling effort performed during the present study. Thus, the non-parametric estimators showed completeness of 58.3% (Chao 2), 64.2% (Jackknife 1) and 53.0% (Jackknife 2) with a mean value of 58.1% suggesting an expected 540 species compared to the actual number recorded in the bay. Even so, the sample-based rarefaction curves suggest that our sampling effort was sufficient to do a good estimation of the theoretical total number of molluscs species from the bay (Fig. 2).

Six sites of the bay registered the higher number of species: Pajarera Island protected coast 1 (50 spp.), Cocinas Island protected coast 2 (48), La Rosada (48) and Cocinas Island protected coast 1 (43), the channel of San Pedro (33) and off the sandy beach of Villa Polinesia (30). Three islets also recorded important numbers: San Andres (44), Mamut (37) and Colorada (34).

The curves of unique and duplicate species in Fig. 2 show that ecological rarity in Bahía de Chamela decreases with increasing sampling effort; this suggests that, although these less frequent species (unique and duplicate) were continuously found during the survey, their incidence became consistently lower throughout the sampling period. The two classes of molluscs with the highest species richness (gastropods and bivalves) had proportionally higher numbers of unique and duplicate species while chitons, scaphopods and cephalopods only one unique species (0.3%) each (Table 3).

Although the 106 species of bivalves were recorded in most sites, 45 (14.6%) were considered as unique (with only one record) and 30 (9.7%) duplicate (recorded in two sites). Almost 25% (75 species) of bivalves contribute to the total rarity (unique + duplicates), therefore it is considered high. Three families included the highest number of species: Veneridae (21), Mytilidae (14) and Carditidae (9); but thirteen families (44.8%), only one species. Most genera (45, 62.5%) had only one species. The gastropods were recorded in all sites of the bay. Three families had the largest number of species Calyptraeidae (18), Columbellidae (19) and Muricidae (19), together representing 30.3% of the gastropods and 18.2% of all mollusc species. However, 30 families (48.4%) had only one species and 12 families (19.4%) two species. Gastropods included 129 (41.9%) unique and 34 (11%) duplicate species.

The revision of the biogeographic affinities shows that most of the species of molluscs of Bahía de Chamela are exclusive to the Realm TEP (299 species, 97.1%); these include many species with wider ranges of distribution towards the two adjacent realms: Temperate Northern Pacific (59 species, 19.2%) and Temperate South America (10, 3.2%). There are a few species also recorded in other tropical realms, such as the eastern and central Indo-Pacific and the American Atlantic, in addition to Temperate Southern Africa and even the Arctic Ocean. Finally, only two species are from PAN and two from COS (Table 4).

Seven species extend their geographic distribution ranges: two bivalves (*Chione tumens* and *Caryocorbula ovulata*) and five gastropods (*Lottia stanfordiana*, *Tegula corteziana*, *Tegula verdispira*, *Anachis adelinae* and *Haminoea vesicula*). The geographic distribution of these species has been documented as follows: *Chione tumens*: Pacific coast of BCS (25^0^N) south to Cabo San Lucas, into the Gulf of California as far as north as Bahia La Choya and Babia San Carlos, Sonora (Huber 2010, Coan and Valentich-Scott 2012); *Caryocorbula ovulata*: Bahia Jianilla, Guanacaste, Coast of Costa Rica to Cabo Blanco, Piura, Peru (Coan and Valentich-Scott 2012); *Lottia stanfordiana*: Head of the Gulf of California south to Guaymas and Espiritu Santo Island, BCS (Keen 1971); *Tegula corteziana*: Described with specimens collected in Cabo Tepoca, Sonora, Mexico (McLean 1970); from north Gulf of California, Cerralvo Island south to Guaymas, Sonora (Keen 1971). These were also recorded in Puerto Peñasco, Sonora (Hendrickx and Brusca 2005). There are also records from Nuevo Vallarta, Nayarit and Bahia Ouhira, Ahome, Sinaloa (NaturaLista https://www.naturalista.mx), but still need to be validated by experts; *Tegula verdispira*: Originally recorded in Maria Magdalena Island, Tres Marias Islands, Mexico. These were also recorded in Bahia Los Frailes, southern end of the Baja California Peninsula (McLean 1970). Pacific coast of BCS (24°N), south to Cabo San Lucas, into the Gulf of California; *Anachis adelinae*: Bahia Magdalena, Baja California, through the Gulf to the Sonoran coast (Keen 1971, Skoglund 2002). Bahia Magdalena BCS, Mazatlan Sinaloa, south to Sayulita, Nayarit (Hendrickx and Brusca 2005); *Haminoea vesicula*: Originally described from shells collected in the southern coast of California (Gould 1855), has also been recorded from Ketchikan, Alaska, through the Coast of Wahington (Dall 1919), California to the coast of the Baja California peninsula and the Gulf of California (Baker and Hanna 1927, Hermosillo et al. 2006).

## Discussion

The inventory of molluscs of Bahía de Chamela includes 12% of the 2,576 species registered in the Mexican Pacific and 6.7% of the 4,643 marine molluscs registered in Mexico (Castillo-Rodríguez 2014). The diversity of molluscs in the bay is similar to and, in many cases, greater than reports from other bays in the TMP and also from other marine protected areas of the TEP of similar extension. Other inventories in the central Mexican Pacific contain fewer species, such as Holguin-Quiñonez and González-Pedraza (1994) with 228 species recorded a much larger area (the coastline of Jalisco, Colima and Michoacán); three inventories focused only on gastropods of certain bays: 1) Sánchez González (1989) with 83 species from Bahia Santiago, Colima, 2) Yáñez-Rivera (1989) with 26 species from Bahia Tenacatita, Jalisco and 3) Esqueda-González et al. (2000) with 70 species from Bahia de Cuastecomate, Jalisco. Further comparisons demonstrate that Bahía de Chamela has much higher species richness of marine molluscs than those recorded in other bays of the tropical Mexican Pacific: Bahia Navachiste (71) (Ortíz-Arellano and Flores-Campaña 2008), Bahias Ohuira and Topobobampo (74) (Corrales-López et al. 2014) and Bahia Guaymas (112) (Hendrickx et al. 2019).

When compared to other marine protected areas of the TEP, the richness of species of Bahía de Chamela is similar to Malpelo Island (393), Sanquianga (356) and Ensenada Utría (316) (UNIVALLE and INVEMAR 2010; López de Mesa and Cantera 2015). The extensive coral reef formations of Malpelo, Sanguianga and Utria are an essential habitat for molluscs that increase the number of species, many exclusive to this particular environment (Cantera et al. 1979, Cosel 1984, Barrios and López 2001). Although the coverage of stony corals in Bahía de Chamela is sparse, there are important aggregations of stony corals *Pocillopora* spp. and *Porites* spp. and the gorgonians *Leptogorgia* spp. and *Pacifigorgia* spp. that offer suitable habitats for the molluscs recorded in the bay associated with these corals (e.g. *Jenneria pustulata*, *Neosimnia avena*, *Simnialena rufa* and *Leiosolenus* spp.).

The 13 chiton species recorded in Bahía de Chamela are included in a single order (Chitonidae) and six families, this being good taxonomic representation for the bay since all intertidal and subtidal species (0-30 m depth) recorded in the TMP belong to the Order Chitonida and are contained in six families. The only genus, not recorded in the present study, was *Lepidozona* which, in the TMP, is represented by *Lepidozona allynsmithi* previously recorded in coral aggregations of Oaxaca (Reyes-Gómez et al. 2010). When compared to other studies, the number of chiton species reported here is similar to the 11 species occurring in Oaxaca (Reyes-Gómez et al. 2010, Flores-Rodríguez et al. 2014) and 13 species in Bahia Acapulco, Guerrero (Galeana-Rebolledo et al. 2012).

The Scaphopoda had a relatively high ecological rarity due to the low number of species recorded in the bay; these have semi-infaunal life forms associated with sandy bottoms with a high level of organic matter where they aggregate. They were collected in sandy bottoms (4-10 m depth) offshore from Villa Polinesia sandy beach and San Andres Islet with a naturalist's trawling dredge. This restricted distribution to particular sites of the bay suggests a preference for habitats probably associated with greater amounts of organic matter and food (e.g. foraminifera). As *Graptacme semistriata* occurs both in the Caribbean and the Eastern Pacific, this may represent a case of sibling species (Steiner and Kabat 2004). Little is known on the scaphopods of the Mexican Pacific due to insufficient studies in this region. Ríos-Jara et al. (2003) recorded 15 species of four genera and two families in silty clay substratum of the continental shelves (72-75 m depth) of Jalisco and Colima (central Mexican Pacific); any of these species coincides with the two found in Bahía de Chamela. Corgos et al. (2014) recorded the highest dominance of scaphopods in areas with finer sediment (e.g. silts and clays) and a higher percentage of organic matter (8-15 m) of Bahia Navidad, Jalisco. Scaphopods accounted for a high percentage of the abundance of macroinvertebrates in Bahia Navidad and, although they do not indicate the species, they point to the genus *Dentalium* as the most frequent.

Two species of cephalopods were recorded in Bahía de Chamela: *Octopus hubbsorum* and *O. bimaculatus*. Records of the Hubbs´s octopus *O. hubbsorum* at five sites indicate the species is well represented in the bay. This is the most common octopus in the Mexican Pacific with an ample distribution from the Gulf of California to Oaxaca. Only one individual of *O. bimaculatus* was found off the sandy beach of Perula during snorkelling at approximately 2 m depth. This is the first record of the species in the ay. Its geographic distribution includes the Northeast Pacific from California (Point Conception) south to Bahia Magdalena on the Pacific coast of the Baja California Peninsula and also from the head of the Gulf of California southwards along the continental coast of Mexico and Central America until Panama (Alejo-Plata et al. 2012, Jereb et al. 2014).

The increase in species reported for Bahía de Chamela is the result of the sampling carried out not only in the intertidal, but also in subtidal habitats with the implementation of different techniques over a long period (2007-2018). Frequently, the species richness of molluscs has been under-estimated due to inadequate coverage of the spatial heterogeneity, as a result of inappropriate sampling techniques, including the failure of the detailed review of soft sediments, macroalgae and even cracks and spaces under the rocks where many species are common. These limitations result in missing specialized species living in limited or specific areas or habitats. Indeed, previous studies on molluscs have pointed out the importance of considering these factors for more complete inventories (Bouchet et al. 2002, Esqueda-González et al. 2014). This survey yielded a substantial increase in the number of species of molluscs (498.4%) compared to those previously recorded in the bay (62 spp.) (Román-Contreras et al. 1991, López-Uriarte et al. 2009). Previous records were again found and are included in the 308 described here. Therefore, 246 species are new records for Bahía de Chamela, including seven species, thus extending their ranges of geographical distribution.

Shallow-water marine mollusc faunas are distributed in a pattern of distinct, geographically-definable areas; their distribution differs strongly and predictably, based on their biogeographic affinities (i.e. species associated with cold, temperate or tropical zones) (Bastida-Izaguirre et al. 2013, Petuch 2013). However, it is not uncommon to find assemblages with different biogeographic affinities. In general, the mollusc assemblage of Bahía de Chamela showed little affinity with realms other than the TEP. Our results are consistent with other studies that also found shallow-water marine mollusc faunas with well-defined biogeographic affinities co-existing in tropical regions where different environments and habitats are available due to environmental heterogeneity (e.g. Bastida-Zavala et al. 2013, Barrientos-Lujan et al. 2017).

The bioregionalization of Spalding et al. (2007) focuses on coastal and shelf waters, combining benthic and pelagic shelf (neritic) biota, which are the areas with the greatest marine biodiversity and where human interest and attention are greatest. It is, therefore, suitable for assigning the affinities of the shallow-water benthic molluscs of Bahía de Chamela and allows recognizing biogeographic patterns and possibly local and regional endemism. Although in general, these molluscs have diverse affinities since they are included in eight of the 12 realms and 10 of the 62 provinces, most of the species are, however, exclusive to a realm (RTEP) and a province (TEP), which indicates homogeneity in the species composition and certain endemism.

In the RTEP, the species composition is likely to be determined by the presence of certain tropical ecosystems and oceanographic/topographic features. The dominant biogeographic forcing agents defining this realm may include isolation, upwelling, nutrient inputs, temperature regimes, currents and coastal complexity. Many studies have demonstrated that the boundaries of shallow-water faunal distribution are correlated to the boundaries of water masses (Castillo-Rodríguez 2014, Stevenson et al. 1998; Culver and Buzas 2000, Benkendorfer and Soares-Gomes 2009). The presence of species with tropical affinities over the RTEP could be explained by the predominance of the North Equatorial Countercurrent (NECC) flowing eastwards between about 4°N and 10°N across the entire Pacific Ocean, transporting warm water from west to east. Approaching the coast of America, the main body of NECC shifts polewards as it flows to the east, parallel to the coast to the north near 5°N and the south near 7°N (Wyrtki 1973). This fact influences the occurrence of warm-water affinity species along the RTEP.

Since the bay is close to the mouth of the Gulf of California, which is the northern boundary region of the RTEP, there are many species shared with the adjacent province Warm Temperate Northeast Pacific (WTNP). Included are some species previously considered endemic to the Gulf of California (i.e. *Lottia stanfordiana*, *Tegula corteziana*) and others that extend their range distribution from the west coast of the Baja California Peninsula and the Gulf of California (i.e. *Chione tumens*, *Anachis adelinae*, *Haminoea vesicula*).

Bahía de Chamela was established as a marine protected area in 2002 because it was recognized that it has a high biodiversity of flora and fauna (CONANP 2008). However, it is not until recent years that detailed studies have been conducted on the marine biota. These studies truly sustain this recognition as an area of high marine biodiversity and coincide in pointing out that the high species richness is related to the coastal geomorphology and the heterogeneity of the seabed (Nuño-Hermosillo et al. 2006, López-Uriarte et al. 2009). Certainly, in the bay, there is an important variety of habitats associated with islands, islets, cliffs, rocky reefs, aggregations of corals, rocky and sandy beaches that increase the heterogeneity and, thus, the availability of marine habitats and, therefore, the variety of benthic microenvironments. In addition, there is a marked seasonal variation throughout the annual cycle in which warm, temperate, dry and rainy seasons are recognized with notable changes in primary and secondary productivity (Silva-Segundo et al. 2008).

The high biodiversity of the bay is also relevant for the conservation of the tropical Mexican Pacific ecosystems, given that over-exploitation of natural habitats and overfishing of some commercial species affect the biodiversity of this region. Conservation strategies must take into account rarity and community-level assessments, including species richness, habitat specificity, reproductive strategies and endemism (Awad et al. 2002, Benkendorff and Davis 2002). In Bahía de Chamela, a large proportion of rare molluscs were found, but only four of these species are currently protected. These are included in the Official Mexican Norm for Endangered Species NOM-059-2010 (SEMARNAT 2010) with a status of conservation: the giant limpet *Scutellastra mexicana*, the mother-of-pearl *Pinctada mazatlanica*, the purple snail *Plicopurpura columellaris* and the cap shell *Crucibulum scutellatum*. They are classified as subject to special protection (Pr) which includes those species that could be threatened by factors that negatively affect its viability and, therefore, it is very important to promote their recovery and conservation. They were recorded in several sampling sites with considerable sizes. Still, we found evidence of its use for self-consumption as food and the sale of its shells as handicrafts or ornaments by fishermen in the bay.

The inventory of species presented here is merely an approximation to the real diversity of molluscs from the bay. Even so, the present work demonstrates that the marine fauna in Bahía de Chamela is well represented by most common families and orders of the five main classes of molluscs (bivalves, gastropods, polyplacophorans, scaphopods and cephalopods). The bay comprises one of the most important protected areas of the tropical Mexican Pacific; this is significant because it displayed high species richness and a large number of unique species. Since the bay is now a popular destination for tourists, efforts to preserve its ecosystems and species are essential. The methodological approaches to estimate the molluscs' diversity must be further improved and more considerable efforts are required in the search for less conspicuous species, those of small sizes, such as micro-molluscs and spec Accept special techniques for the search for very particular life forms, such as nudibranchs or stenotic species that are symbionts of other invertebrates and fish. The information about molluscs in Bahía de Chamela should be complemented with an analysis that includes an assessment of the α, γ and β diversity to determine their relative distribution on different spatial scales. Population studies are also required, in particular of the species of commercial interest, which contribute to an integral framework on the biology and ecology of these species which is essential for their conservation in the bay and throughout its range of distribution.

## Supplementary Material

1E4C6F28-E2FE-5092-A280-BC12BFB93771 10.3897/BDJ.8.e59191.suppl1 Supplementary material 1 Checklist of marine molluscs of Bahía Chamela with information on their local distribution and biogeographic affinities. Data type Checklist of species, local distribution and biogeographic affinities. Brief description Checklist of marine molluscs of Bahía Chamela with information on their local distribution in Bahia de Chamela, Mexico and the biogeographic affinities. Location and geographic position of the sampling sites in Table 1. Biogeographic classification based on the spatial units (Realms, Provinces, Ecoregions) proposed by Spalding et al. (2007). File: oo_475113.xlsx https://binary.pensoft.net/file/475113 Eduardo Ríos-Jara, Cristian Moisés Galván-Villa, María del Carmen Esqueda-González, Manuel Ayón-Parente, Fabián Alejandro Rodríguez-Zaragoza, Dafne Bastida-Izaguirre, Adriana Reyes-Gómez.

## Figures and Tables

**Table 1.** Location and geographic position of the sampling sites in Bahía de Chamela. The method used to obtain the samples and the depth of each site is also shown. TD = naturalist´s trawling dredge, SK = direct search during snorkelling, SD = direct search during SCUBA diving, SI = direct search in the intertidal.

Sampling sites	Location	Latitude / Longitude	Depth (m)	Sampling method
1	Off the sandy beach of Perula 1	19°31'34.5''N, 105°5'31.3''W	7	TD
2	Off the sandy beach of Perula 2	19°33'23.6''N, 105°6'20.0''W	8	TD
3	Off the sandy beach of Perula 3	19°33'20.7''N, 105°6'22.3''W	5	TD
4	Villa Polinesia	19°34'5.9''N, 105°6'7.1''W	7	TD
5	Off San Pedro Island	19°32'4.0''N, 105°5'5.0''W	7	TD
6	Channel between Cocinas and Pajarera	19°33'13.2''N, 105°6'37.7''W	8	SD
7	Channel of San Pedro Island	19°32'1.0''N, 105°5'17.9''W	9	SD
8	El Novillo Islet	19°33'22.6''N, 105°6'31.1''W	7	SD
9	Embarcadero	19°31'50.4''N, 105°4'57.0''W	3	SD
10	Estero Pérula	19°35'18.3''N, 105°8'7.8''W	0.5	SI
11	Off Pajarera Island	19°33'39.9''N, 105°6'46.5''W	5	SD
12	Mamut Islet	19°32'57.5''N, 105°6'50.3''W	12	SD
13	Cocinas Island exposed coast 2	19°32'52.1''N, 105°6'48.6''W	9	SD
14	Cocinas Island protected coast 1	19°33'7.9''N, 105°6'31.8''W	6	SD
15	Cocinas Island protected coast 2	19°32'45.7''N, 105°6'27.7''W	8	SD
16	Cocinas Island protected coast 3	19°32'57.5''N, 105°6'20.3''W	12	SD
17	Colorada Islet	19°32'23.9''N, 105°5'31.9''W	8	SD
18	Pajarera Island exposed coast 1	19°33'27.7''N, 105°7'0.6''W	15	SD
19	Pajarera Island exposed coast 2	19°33'19.5''N, 105°7'2.2''W	25	SD
20	Pajarera Island protected coast 1	19°33'29.3''N, 105°6'40.2''W	4	SD
21	Pajarera Island protected coast 2	19°33'22.9''N, 105°6'50.1''W	8	SD
22	Pajarera Island protected coast 3	19°33'44.3''N, 105°6'42.2''W	10	SD
23	San Agustin Islet	19°32'8.4''N, 105°5'15.54''W	5	SD
24	San Andrés Islet	19°31'32.9''N, 105°5'31.8''W	8	SD
25	San Pedro Island exposed coast	19°31'53.5''N, 105°5'22.3''W	10	SD
26	La Negrita Islet	19°31'19.9''N, 105°5'5.8''W	8	SD
27	La Rosada	19°33'1.6''N, 105°5'39.0''W	13	SD
28	Los Anegados Islet	19°34'35.8''N, 105°7'59.7''W	6	SD
29	Los Negritos	19°31'35.9''N, 105°4'59.9''W	1	SI
30	Punta Chamela	19°31’50.4''N, 105°4'59.1''W	2	SI
31	Roca Perula	19°34'48.9''N, 105°7'58.8''W	3	SK, SD

**Table 2.** Summary of the number and percentages of species, genera, families and orders of the five classes of molluscs recorded in Bahía de Chamela. The percentages represent the proportion of the total value of the same taxa for all mollusc groups.

Taxon	Bivalvia	Gastropoda	Polyplacophora	Scaphopoda	Cephalopoda	Total
Species	106 (34.4%)	185 (60.1%)	13 (4.2%)	2 (0.6%)	2 (0.6%)	308
Genera	72 (34.8%)	123 (59.4%)	9 (4.3%)	2 (1.0%)	1 (0.5%)	207
Families	29 (29.0%)	63 (63.0%)	6 (6.0%)	1 (1.0%)	1 (1.0%)	100
Orders	14 (45.2%)	14 (45.2%)	1 (3.2%)	1 (3.2%)	1 (3.2%)	31

**Table 3.** The ecological rarity of molluscs species in 31 sampling sites of Bahía de Chamela, México. The percentages refer to the proportion of unique and duplicated species to the total number of species of molluscs recorded.

Rarity	Bivalvia	Gastropoda	Polyplacophora	Scaphopoda	Cephalopoda	Total
Unique	45 (14.6%)	129 (41.9%)	1 (0.3%)	1 (0.3%)	1 (0.3%)	179 (57.9%)
Duplicate	30 (9.7%)	34 (11.0%)	2 (0.6%)	1 (0.3%)	0	67 (21.8%)

**Table 4.** Biogeographic affinities documented for the mollusc species of Bahía de Chamela, Mexico. The percentages refer to the proportion of species in a particular region to the total number of species recorded in the bay (308). * Five species, previously considered endemic to the Gulf of California, extend their distribution ranges south to Bahía de Chamela.

Biogeographic region	Number of species
Realm Tropical Eastern Pacific (RTEP)	299 (97.1%)
Province Tropical East Pacific (TEP)	296 (96.1%)
Ecoregion Tropical Mexican Pacific (TMP)	7 (2.3%)
Tres Marias Islands, Mexico (ILTM)	21 (6.8%)
Ecoregion Revillagigedo Islands, Mexico (IREV)	11 (3.6%)
Ecoregion Clipperton Atoll (ICLI)	2 (0.6%)
Ecoregion Coco Island, Costa Rica (ICOC)	18 (5.8%)
Province Galapagos Islands, Ecuador (IGAL)	43 (14.0%)
Realm Temperate Northern Pacific (RTNP)	59 (19.2%)
Province Warm Temperate Northeast Pacific (WTNP)	56 (18.2%)
Ecoregion Corteziana (COR)	28* (9.1%)
Realm Temperate South America (RTSA)	10 (3.2%)
Province Warm Temperate Southeastern Pacific (WTSP)	1 (0.3%)
Realm Central Indo-Pacific (RCIP)	1 (0.3%)
Province Western Coral Triangle (WCT)	1 (0.3%)
Realm Eastern Indo-Pacific (REIP)	1 (0.3%)
Province Hawaii (HA)	1 (0.3%)
Realm Tropical Atlantic (RTA)	6 (1.9%)
Province Tropical Northwestern Atlantic (TNA)	5 (1.6%)
Province North Brazil Shelf (NBS)	4 (1.3%)
Province Tropical Southwestern Atlantic (TSA)	2 (0.6%)
Realm Temperate Southern Africa (RTSAF)	1 (0.3%)
Province Agulhas (AG)	1 (0.3%)
Realm Artic (RAR)	1 (0.3%)
Pantropical (PAN)	2 (0.6%)
Cosmopolite (COS)	2 (0.6%)
